# Chemotherapy treatment induces an increase of autophagy in the luminal breast cancer cell MCF7, but not in the triple-negative MDA-MB231

**DOI:** 10.1038/s41598-017-07489-x

**Published:** 2017-08-03

**Authors:** Christian Garbar, Corinne Mascaux, Jérôme Giustiniani, Yacine Merrouche, Armand Bensussan

**Affiliations:** 1 Biopathology Department, Institut Jean Godinot–Unicancer, 1 rue du Général Koenig CS80014, 51726 Reims, France; 2 Oncology Department, Institut Jean Godinot–Unicancer, 1 rue du Général Koenig CS80014, 51726 Reims, France; 3 Research Department, Institut Jean Godinot–Unicancer, 1 rue du Général Koenig CS80014, 51726 Reims, France; 4 DERM-I-C EA7319, Université de Reims Champagne Ardenne, 51 rue Cognacq Jay, 51095 Reims, France; 5 Institut National de la Santé et de la Recherche Médicale (INSERM) U976, Hôpital Saint Louis, 75010 Paris, France; 6 Université Paris Diderot, Sorbonne Paris Cité, Laboratoire Immunologie Dermatologie & Oncologie, UMR-S 976, 75475 Paris, France

## Abstract

Autophagy is one of the chemotherapy resistance mechanisms in breast cancer. The aim of this study was to determine the level of recruitment of the autophagy pathway in the triple-negative breast cancer (TNBC) cell line MDA-MB231 compared with that in the control luminal breast cancer cell line MCF7 before and after treatment with chemotherapy drugs. Furthermore, we investigated the relationship between autophagy and EGFR, MUC1 and IL17-receptors as activators of autophagy. Immunohistochemistry was performed in cell culture blocks using LC3b, MUC1-C, EGFR, IL17A, IL17-RA and IL17-RB antibodies. We found that the basal autophagy level in MDA-MB231 was high, whereas it was low in MCF7. However, in contrast to MDA-MB231, the autophagy level was increased in MCF7 upon treatment with chemotherapy agents. Interestingly, we observed that the expression levels of MUC1-C, EGFR, IL17-RA, and IL17-RB were not modified by the same treatments. Furthermore, the chemotherapy treatments did not increase autophagy in TNBC cells without affecting the expression levels of MUC1-C, EGFR, IL17-RA or IL17-RB.

## Introduction

Perou’s biological and clinical classification of breast cancers (BCs) was proposed by the St Galen International Expert Consensus and is currently widely used in the clinic. This classification system proposes the following three main molecular subtypes: luminal (LUM) BC, which expresses hormonal estrogen and progesterone receptors (ER+ and PR+) but no human epidermal growth receptor 2 (HER2-); overexpressed HER2 BC (ER+/− PR+/− HER2+); and triple negative (TN) BC, which lacks these receptors HER2, ER and PR^1, 2^.

LUM BC benefits from anti-estrogen therapy, such as tamoxifen, which is an aromatase inhibitor, and HER2 BC is treated with targeted anti-HER2 (i.e., trastuzumab) therapy. Treatments of TN BC remain more challenging and are mainly based on cytotoxic chemotherapy, such as docetaxel, cyclophosphamide, fluorouracil, and epirubicine^3–5^.

Autophagy is an adaptive cellular mechanism to external stress and contributes to cell survival and homeostasis. Autophagy is a complex pathway involving multiple proteins. First, autophagosomes consisting of an isolated membrane in the cytosol are activated by class I PI3K and Atg complexes, leading to nucleation, which involves Beclin-1. Finally, LC3 proteins (microtubule-associated protein 1 light chain 3) control the elongation phase, which stabilizes the autophagosomes. LC3 proteins are stable and persistent and are widely used to monitor autophagy^6^. Tumor cells exhibit a basal level of autophagy activity that could be increased upon exposure to anoxia, starvation, chemical agents and radiation.

Chemotherapy resistance is due to multiple mechanisms, including autophagy. For example, in breast cancer, epirubicin reduces autophagy and protects cells from chemotherapy-induced apoptosis. Moreover, in colorectal cancer, oxaliplatin and 5-fluorouracil were found to have an improved efficiency in the presence of an anti-autophagy agent, whereas in lung cancer, anti-EGFR agents (i.e., gefitinib or erlotinib) activate autophagy and induce drug resistance^7^.

MUC1 is a large transmembrane O-glycosylated heterodimer protein that consists of a large, broadly glycosylated extracellular α-subunit containing 20 to 125 tandem repeats of 20 amino acids (MUC1-VNTR) and a β-subunit containing the transmembrane domain and a cytoplasmic tail (MUC1-C)^8–10^. The β-subunit is involved in several cellular signaling pathways, such as growth/survival pathways and the induction or inhibition of apoptosis^11, 12^. Moreover, MUC1 expression is associated with an increased lysosomal turnover of the autophagy maker LC3 after stimulation of the AMP-activated protein kinase (AMPK), which is involved in the regulation of autophagy^13^. Some authors have demonstrated that the β-subunit of MUC1 and EGFR are both co-localized in the cell membrane and nucleus and are involved in the internalization of EGFR and the activation of the EGFR-PI3K-AKT-mTOR pathway^14–17^. Interestingly, this pathway is a key regulator of autophagy, and activated mTOR inhibits autophagy^7^.

ERK1/2 is an autophagy activator. Additionally, our team has recently shown that IL17A is produced by BC TILs and plays a role in docetaxel chemoresistance and proliferation through the ERK1/2 pathway^18^. We also reported that IL17A and IL17B receptor transcripts are overexpressed in BCs and that the activation of the IL17E receptor, i.e., the heterodimer of IL17A and IL17B receptors, induces EGFR phosphorylation and migration to the nucleus. We have also suggested that the inhibition of IL17E could enhance the efficacy of anti-EGFR chemotherapy^19, 20^.

To better understand the relationship between autophagy and the chemotherapy resistance of TN BC, we used a cell culture model consisting of a TNMDA-MB231 cell line and an MCF7 LUM control cell line with or without a treatment with sub-lethal concentrations of chemotherapy agents. We then performed immunohistochemistry to measure the expression levels of LC3b, which is an autophagy marker, and the targeted antigens MUC1, EGFR, IL17RA and IL17RB, which are known to be involved in autophagy and chemoresistance.

## Results

### Basal autophagy level is high in MDA-MB231 cells and is not influenced by chemotherapy drugs

**Figure 1:**
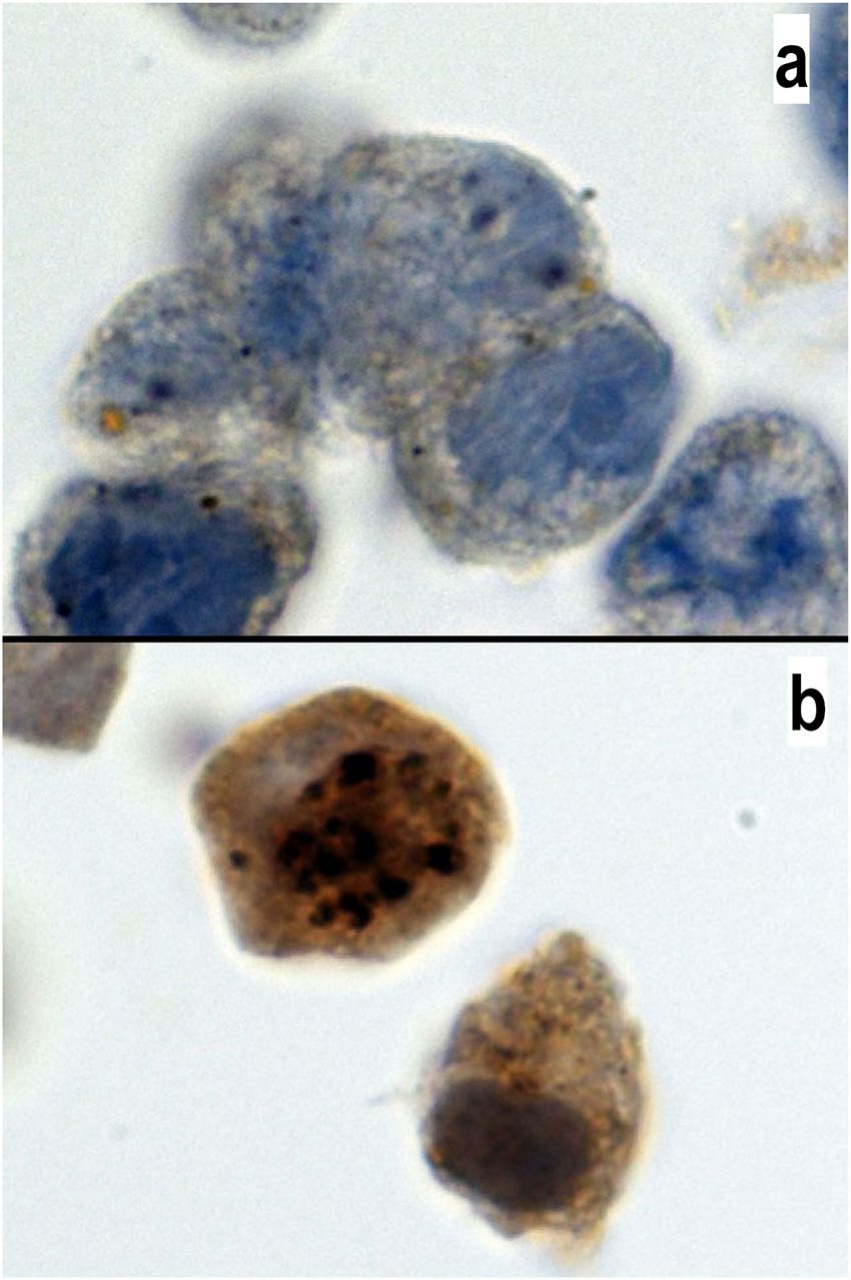
Expression of LC3b in MCF7 and MDA-MB231 cells. LC3b antibody staining in MCF7 (**a**) and MDA-MB231 (**b**) cells. Positive staining is characterized by a cytoplasmic dot signal. Note the low basal level in MCF7 cells and the high basal level in MDA-MB231 cells. The LC3b antibody was diluted 1/400 (100x magnification).

**Figure 2:**
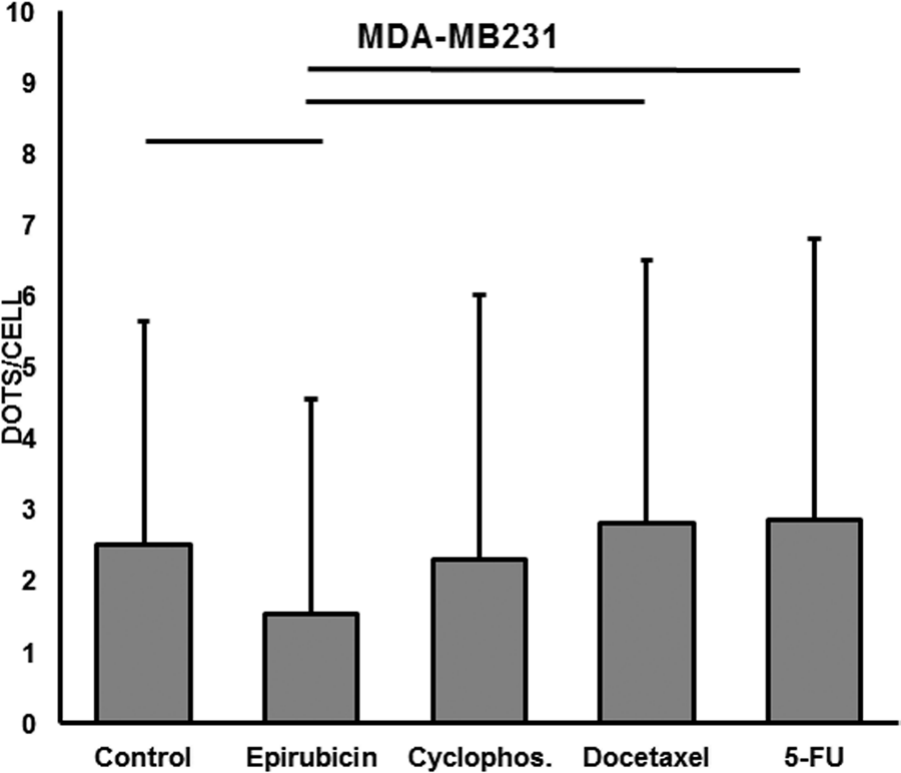
Quantification of autophagy in MDA-MB231. Mean and SD of the cytoplasmic dot signals of LC3b in 100 cells from each MDA-MB231 culture. Line illustrates a p-value <0.05 (Mann-Whitney test). Significant differences were observed between the control and epirubicin-, docetaxel- or 5-FU-treated cultures.

The MDA-MD231 cell line consists of triple-negative breast cancer cells that do not express estrogen and progesterone receptors or HER2. These characteristics are conserved in the cells upon exposure to chemotherapy agents (data not shown). When cultured in the control medium, LC3b staining was higher in the in MDA-MD231 cells than in the MCF7 cells (Fig. 1). Compared with cells cultured in the control medium, cells cultured with epirubicin exhibited a significantly decreased basal level of autophagy. Importantly, the autophagy level in the MDA-MD231 cells cultured with cyclophosphamide, docetaxel and 5-FU was similar to that in the MDA-MD231 cells cultured in the control culture (Fig. 2).

### Basal autophagy is low and increases with chemotherapy drugs in MCF7 cells

**Figure 3:**
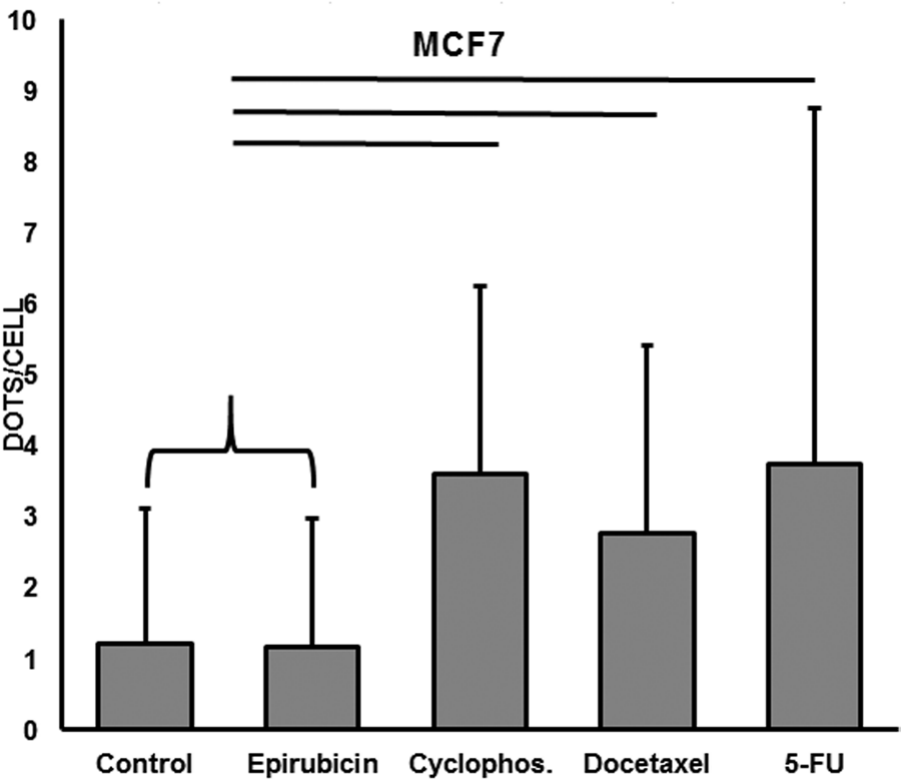
Quantification of autophagy in MCF7 cells. Mean and SD of cytoplasmic dot signals of LC3b in 100 cells from each MCF7 culture. Line indicates p <0.05 (Mann-Whitney test). Significant differences were observed between the control and cyclophosphamide, docetaxel, 5-FU or epirubicin treated cells.

MCF7 cells are a luminal cancer cell line expressing estrogen and progesterone receptors but not HER2. These characteristics were conserved upon exposure to chemotherapy agents (data not shown). Interestingly, as shown in Fig. 3, MCF7 cells treated with the autophagy inhibitor epirubicin still had the same low basal level of autophagy as the MCF7 cells cultured in the control medium (control medium MCF7 vs control medium MDA-MB231: p = 0.006, control medium MCF7 vs epirubicin MDA-MB231: p = 0.45) and that treatments with cyclophosphamide, docetaxel and 5-fluorouracil (5-FU) significantly enhance the level of autophagy.

### Expression levels of MUC1, EGFR, IL17-RA and IL17-RA are not influenced by chemotherapy

**Figure 4:**
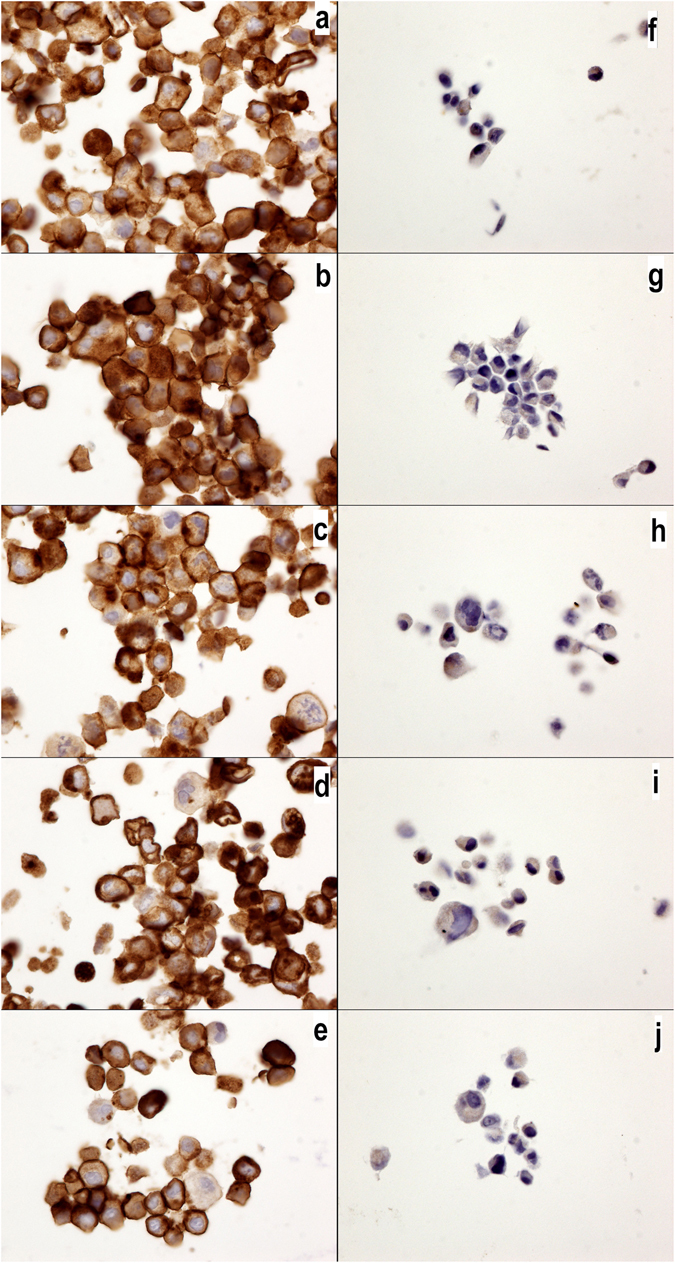
MUC1-C expression in MCF7 and MDA-MB231 cells. MUC1-C expression in MCF7 (**a**–**e**) and MDA-MB231 cells (**f**–**j**) without drugs or (**a**,**f**) with cyclophosphamide (**b**,**g**), doxorubicin (**c**,**h**), epirubicin (**d**,**i**) or 5-fluorouracil (**e**,**j**). Note the higher expression of MUC1-C in the MCF7 cells than that in the MDA-MB231 cells, but no changes were observed with the chemotherapy drug treatments. The MUC1-C antibody was diluted 1/400 (40x magnification).

**Figure 5:**
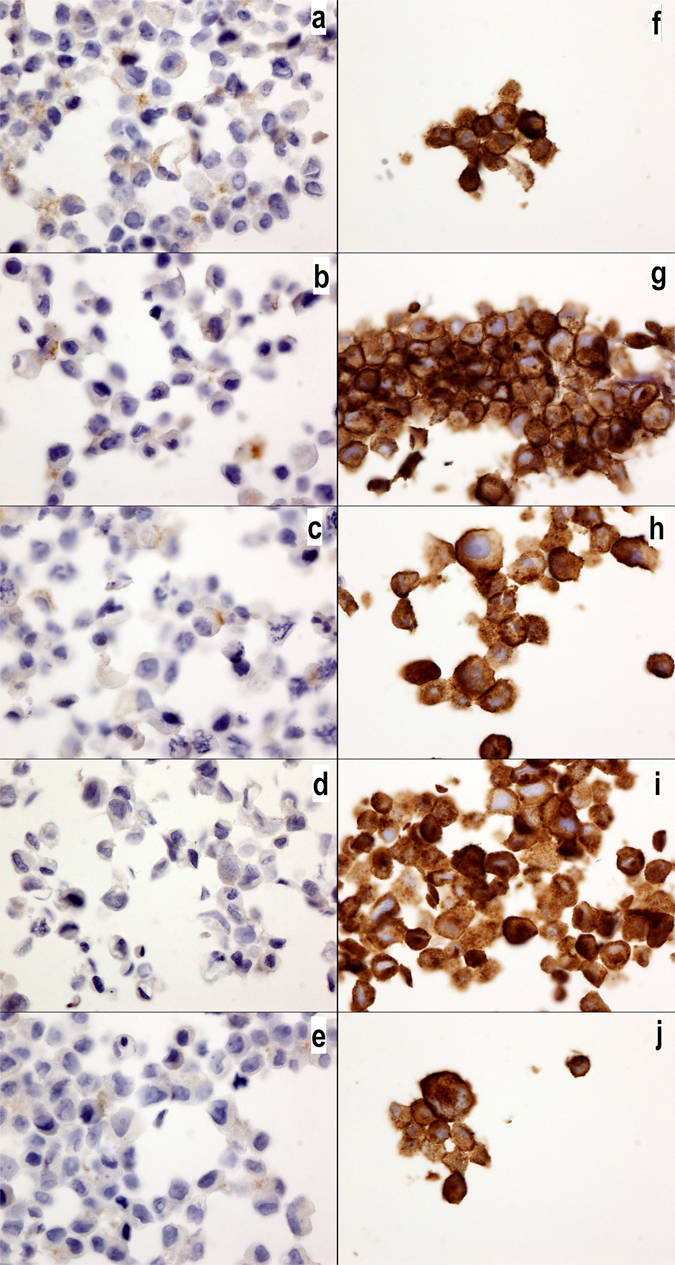
EGFR expression in MCF7 and MDA-MB231 cells. EGFR expression in MCF7 (**a**–**e**) and MDA-MB231 cells (**f**–**j**) without drugs or (**a**,**f**) with cyclophosphamide (**b**,**g**), doxorubicin (**c**,**h**), epirubicin (**d**,**i**) or 5-fluorouracil (**e**,**j**). Note the higher expression of EGFR in the MDA-MB231 cells than that in the MCF7 cells, but no changes were observed with the chemotherapy drug treatment. The EGFR antibody was diluted 1/200 (40x magnification).

We have recently described that luminal breast cancer cells widely express MUC1 and exhibit a low level of EGFR *in situ*, whereas triple-negative breast cancer cells are negative for MUC1 and positive for EGFR^21^. Here, we confirmed these results using the luminal breast cancer cell line MCF7 and the triple-negative breast cancer cell line MDA-MB231. Interestingly, we observed no differences in the expression of MUC1 or EGFR in the MCF7 or MDA-231 cell cultures treated with the control medium alone, the autophagy inhibitor epirubicin, or chemotherapy drugs (Figs 4 and 5).

**Figure 6:**
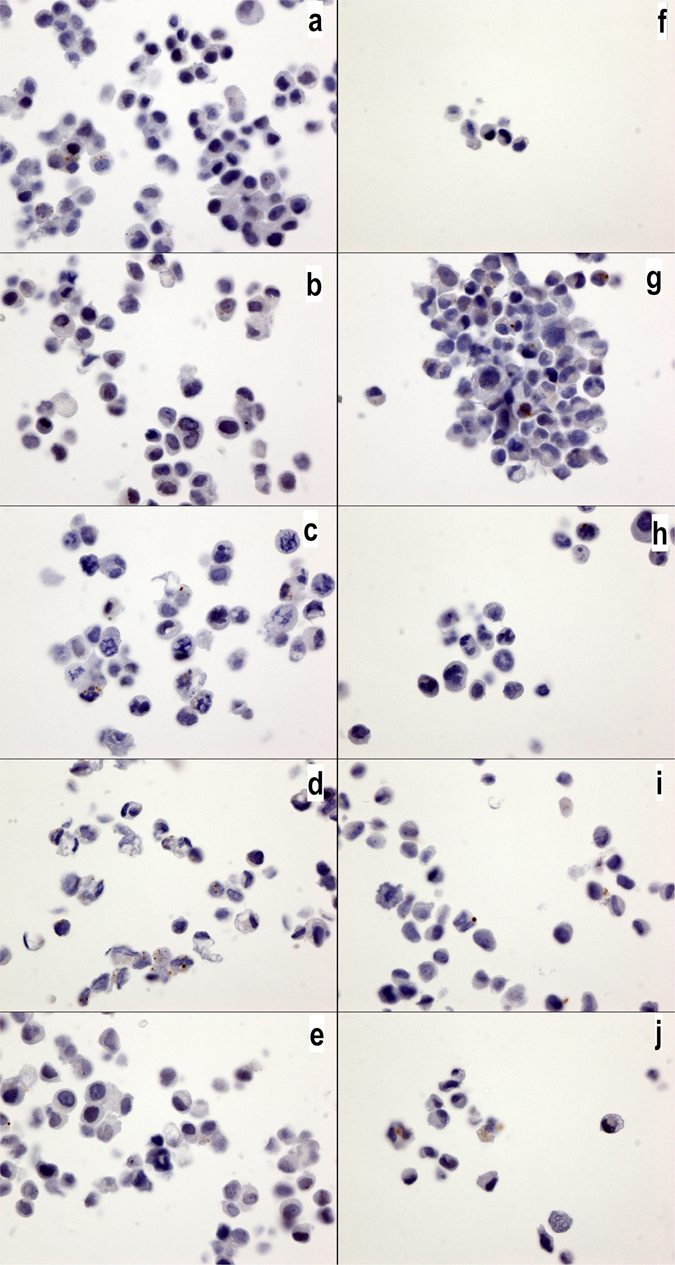
IL17A expression in MCF7 and MDA-MB231 cells. IL17A expression in MCF7 (**a**–**e**) and MDA-MB231 cells (**f**–**j**) without drugs or (**a**,**f**) with cyclophosphamide (**b**,**g**), doxorubicin (**c**,**h**), epirubicin (**d**,**i**) or 5-fluorouracil (**e**,**j**). No significantly positive signals were observed in any of the cultures. The IL17a antibody was diluted 1/800 (40x magnification).

**Figure 7:**
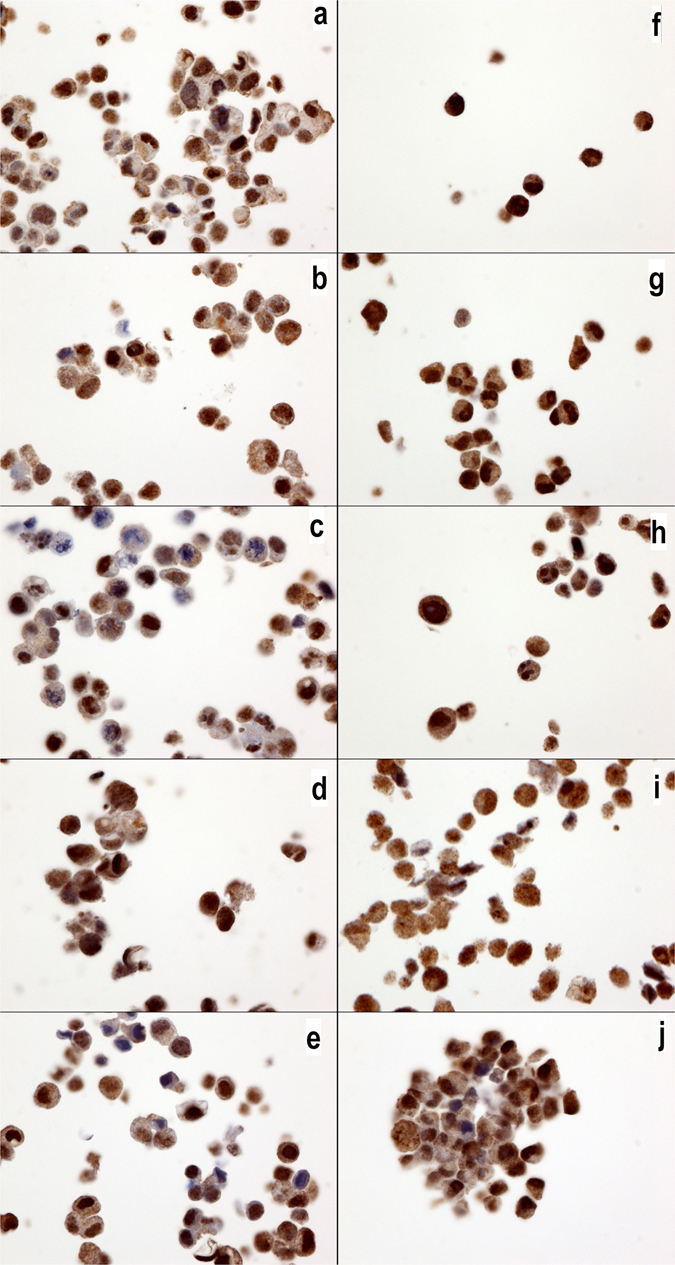
IL17RA expression in MCF7 and MDA-MB231 cells. IL17RA expression in MCF7 (**a**–**e**) and MDA-MB231 cells (**f**–**j**) without drugs or (**a**,**f**) with cyclophosphamide (**b**,**g**), doxorubicin (**c**,**h**), epirubicin (**d**,**i**) or 5-fluorouracil (**e**,**j**). All cell cultures show a positive cytoplasmic signal, but no changes were observed with the chemotherapy drug treatments. The IL17RA antibody was diluted 1/800 (40x magnification).

**Figure 8:**
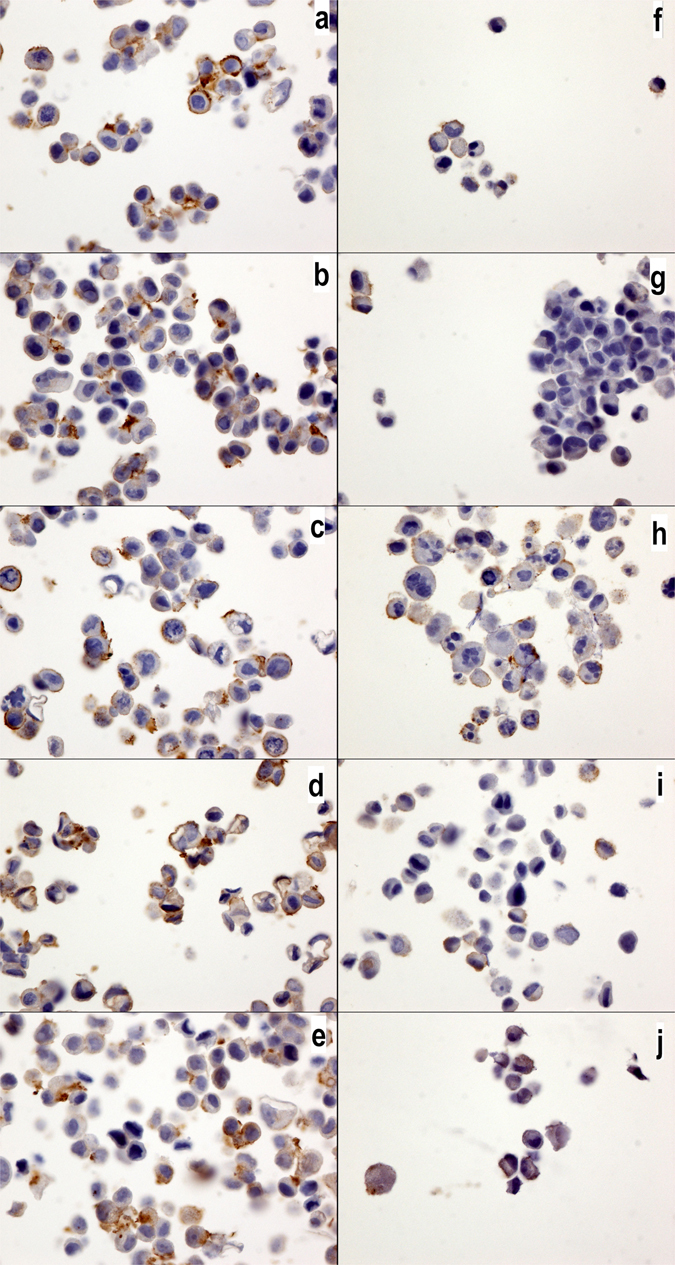
IL17RB expression in MCF7 and MDA-MB231 cells. IL17RB expression in MCF7 (**a**–**e**) and MDA-MB231 cells (**f**–**j**) without drugs or (**a**,**f**) with cyclophosphamide (**b**,**g**), doxorubicin (**c**,**h**), epirubicin (**d**,**i**) or 5-fluorouracil (**e**,**j**). All cells cultures exhibited a positive membrane signal, but no changes were observed with the chemotherapy drug treatments. Note that the MCF7 cells show a higher expression than the MBA-MB231 cells. The IL17RB antibody was diluted 1/40 (40x magnification).

IL17A is known to inhibit autophagy^22^. We have previously demonstrated that IL17A transcripts are not detected in breast cancer cell lines, although these cell lines do express IL17RA and IL17RB transcripts^19^. In the present work, specific antibodies were used for the first time, and we confirmed our previous results: IL17A is not expressed in any culture cells (Fig. 6) comparatively to IL17-RA (Fig. 7). Moreover, we also observed a lower level of IL17RB in the MDA-MD231 cells than in the MCF7 cells (Fig. 8). Interestingly, the expression of IL17RA and IL17RB was not influenced by the chemotherapy drugs (Figs 7 and 8), and IL17A remained negative (Fig. 6)

## Discussion

In the present study, we performed an analysis investigating the recruitment of the autophagy pathway in triple-negative cancer cells. The luminal cancer cells showed a low level of autophagy, which was increased by chemotherapy drugs. However, epirubicin, which is a known autophagy inhibitor, failed to induce this enhancement^7^. By contrast, the triple-negative cancer cells showed a high level of autophagy, which was not influenced by the chemotherapy drugs. Interestingly, epirubicin reduced the basal level of autophagy.

This high level of autophagy in TN BC has already been observed *in vitro* and *in vivo* by Lefort S. *et al*. in a large cohort of BC cells. These authors demonstrated that TN BC shows significantly more autophagosomes (measured by LC3b) than LUM or HER2 BC. Furthermore, these authors demonstrated that the LC3b protein is a marker of a poor prognosis in TN BC patients using immunohistochemistry on paraffin embedded biopsy tissue^23^. Using a mouse model, chloroquine, which is an autophagy inhibitor, was demonstrated to potentiate chemotherapy against human TN BC^23^.

The aim of our present work was to establish a kinetic cell culture model to better understand chemoresistance. We found that TN BC (MDA-MB231) and LUM (MCF7) cells exhibit a different strategy against chemotherapy drugs. TN BC has an already high initial level of autophagy, whereas LUM exhibits increased autophagy in response of chemotherapy. These cell strategy differences could be explained by the dual paradoxical role of autophagy against therapeutic anticancer drugs, which either induce cell death or promote cell survival^7^. It is easy to understand that treatment against autophagy will yield different results in the same tumors and across tumor subtypes, leading to a true targeted therapy.

The ERK1/2 signaling pathway is a key component of autophagy. Sivaprasad U *et al*. demonstrated that TNF activates autophagy in MCF7 BC and that the pharmacological inhibition of ERK1/2 was associated with a decrease in TNF-induced autophagy (LC3b)^24^. In similar cell models using MCF7 and MDA-MB231 cell lines, Shen P. *et al*. demonstrated that gemcitabin induces autophagy with a cytotoxic effect in MCF7 cells and a cytoprotective effect in MDA-MB231 cells. The cytotoxic effect in MCF7 cells is attributed to the cascade of estrogen receptor-ERK-p62, which could be inactivated by specific siRNA. Furthermore, these authors showed that gemcitabin is able to induce mTOR-independent autophagy in MDA-MB231 cells also via the ERK1/2 pathway^7, 25, 26^.

Interestingly, our team demonstrated that IL17A promotes chemoresistance to docetaxel and proliferation through ERK1/2 signaling in the MCF7, T47D, BT20, MDA468 and MDA157 cell lines^18, 19^. We also showed that IL17A is produced by tumor-infiltrating lymphocytes isolated in biopsies from six patients with TN BC. The mechanism of this chemoresistance is not currently understood, but inhibition of the autophagy pathway in TN BC is likely the best candidate. Indeed, Zhou Y. *et al*., using a hepatocellular carcinoma cell culture model, demonstrated that IL17A promotes the migration of tumor cells and prevents autophagic cell death^22^. Our previous studies also demonstrated that MCF7 and MDA-MD231 cells do not express IL17A mRNA, but do express the transcripts of its receptor IL17RA. Furthermore, we found that MCF7 cells expressed IL17RB mRNAs, whereas MDA-MB231 cells only expressed a low level of IL17RB mRNA^19^. The present study confirmed these observations using immunohistochemistry. We recently described the possible synergy between EGF and IL17RE to confer resistance against anti-EGFR therapy^20^. Here, we illustrated that protein expression of IL17RA, IL17RB, EGFR and MUC1 is not altered by chemotherapy and, consequently, that these proteins could be accessible for specific targeted therapy. It should be noted that in this study, we also confirmed *in vitro* our previous *in vivo* observations indicating that TN BC cells present a low level of MUC1 and a high level of EGFR and that LUM BC cells show the opposite pattern^21^. Moreover, MUC1 expression is correlated with an increased lysosomal turnover of the autophagic maker LC3, suggesting that MUC1 plays a role in the regulation of autophagy^27^.

Recently, Hiraki M *et al*. demonstrated, using the TN BC cell line MDA-MB468, that MUC1-C activated the MEK-ERK and PI3K-AKT pathways, and both activated autophagy. These authors concluded that targeting MUC1-C is a potential strategy for reversing resistance in TN BC^28^. We do not completely agree with this hypothesis because in our experience, both *in vivo and in vitro*, TNBC shows no or low levels of MUC1-C^21^. Another study using MCF7 and ZR-75-1 cell lines treated with an inhibitor of MUC1-C (GO-203) demonstrated an increase in the apoptotic response to Taxol and doxorubicin^29^. It is known that the MUC1-C cytoplasmic domain interacts with numerous regulator proteins, such as NF-B, p53 and PI3k^30^. As discussed above, the PI3K-AKT-mTOR pathway is involved in autophagy. Recently, we have demonstrated that PI3K-p110β and MUC1-C are more highly expressed in LUM than in TN BC^21^. This finding suggests that BC cell autophagy activation strategies could involve the MUC1-PI3K-AKT pathway in LUM BC and the IL17-ERK pathway in TN BC.

In conclusion, this study illustrated the different strategies of inducing autophagy in MDA-MB231 and MCF7 cells in response to chemotherapy. Further studies should be performed to confirm our hypothesis and identify the ideal strategy for anti-autophagy adjuvant therapy to avoid chemotherapy resistance in each BC subtype.

## Materials and Methods

### Cell cultures and reagents

The MCF7 and MDA-MB231 cell lines were obtained from England Type Culture Collection (Salisbury). The cells were cultured in DMEM medium (VWR International SAS, Fontenay-sous-bois, France) supplemented with 10% Fetal Calf Serum (FCS), 2% glutamine and 1% penicillin/streptomycin antibiotics (Dutscher SAS, Brumath, France). All cells were kept at 37 °C in a 5% CO2 atmosphere incubator. Then, each cell line was incubated with a sub-lethal chemotherapy drug concentration calculated according to the mean of the metabolic activity as measured by Rotitest Vital® (Carl Roth EURL, Lauterbourg, France) and a spectrophotometer (iMark TM, Bio-Rad, France) as follows: the final concentrations consisted of only DMEM in the control culture, 5 µg/ml in the epirubicin culture, 10 µg/ml in the docetaxel culture, 10 µg/ml in the 5-fluorouracil culture and 10 µg/ml in the cyclophosphamide culture. The chemotherapy drugs were added, and cell blocks were performed the following day. Each cell culture was replicated 3 times for the cell blocks and immunohistochemistry.

### Cell block

After washing with PBS and treating with trypsin, the cell cultures were centrifuged at 1300 RPM for 8 min. Then, the cell pellets were fixed in 5 ml of 4% buffered formaldehyde solution for 8 to 48 hours. The cells were centrifuged at 1000 RPM for 5 min. The pellets were prepared using a Cytoblock® kit (Thermo Fisher Scientific, Brebieres, France) and then embedded in paraffin. The paraffin-embedded cell blocks were cut into 4 µm thick sections for the immunohistochemistry.

### Immunohistochemical Methods

Immunohistological staining was performed using a Dako Autostainer Link 48® immunostaining system (Dako Glostrub, Denmark). After dewaxing, the antigenic retrieval was performed using citrate buffered (pH 6) or EDTA buffered (pH 9) antigenic retrieval solution at 99 °C in a warm bath (EnVision Flex Target Retrieval solutions with a high and low pH, Dako). Endogenous peroxidase was inhibited with a hydrogen peroxide phosphate buffered solution (EnVision Flex Peroxidase Blocking Reagent, Dako). After incubating with the primary antibodies, the immunological reaction was revealed by a polymer dextran coupled with the secondary antibody and peroxidase for 15 min (EnVision Flex HRP, Dako) and diaminobenzidine for 10 minutes (EnVision DAB+ chromogen, Dako). Counterstaining was performed with hematoxylin for 10 min (EnVision Flex hematoxylin, Dako). Negative controls were obtained using mouse or rabbit IgG1 (Universal Negative Control Mouse or Universal Negative Control Rabbit, Dako) instead of the primary antibodies for each culture. No specific signal was observed. The primary antibodies, dilutions and antigenic retrieval are described in Table 1 (Table 1).

**Table 1:** Primary antibodies, dilution, antigenic retrieval, incubation times and abbreviations used in this study.

Antibodies	Clone	Abbreviation	Manufacture	Dilution	Retrievial	Incubation (minutes)
EGFR wild-type	DAK-H1-WT	EGFR	Dako	1: 200	pH9	30
MUC1-ter C	ARP41446	MUC1-CT	Aviva Syst. Biology	1: 400	pH9	60
α- Estrogen receptor	SP1	ER	Dako	RTU	pH9	20
Pregesteron receptor	PgR636	PR	Dako	RTU	pH9	20
HER2	c-ErB2	HER2	Dako	1: 800	pH6	30
LC3b	poly	LC3b	Abcam	1: 400	pH6	60
IL17a	poly	IL17A	Abcam	1: 800	pH6	60
IL17RA	49M4D2	IL17RA	Clniscience	1: 800	pH6	60
IL17RB	97C691	IL17RB	Cliniscience	1: 40	pH6	60

### Immunostaining quantification

The staining results were evaluated by C.G. and C.M. Only LCB3 was quantified and is expressed as the mean and SD of the cytoplasmic dots signals (autophagosomes) in 100 cells at 100x magnification (Fig. 1). Other immunohistochemical staining was evaluated as positive or negative.

### Statistics

The results are expressed as the means and standard error. Mann-Whitney tests were performed. A p-value <0.05 was considered significant. The WinSTAT® version 2012 (Fitch Software, Bad Krozinger, Germany) and Excel 2013 (Microsoft Corp., Redmond, Washington U.S.A.) programs were used for the statistical analysis.
